# Measuring Intolerance of Uncertainty After Acquired Brain Injury: Factor Structure, Reliability, and Validity of the Intolerance of Uncertainty Scale–12

**DOI:** 10.1177/10731911231182693

**Published:** 2023-06-26

**Authors:** Andrea Kusec, Fionnuala C. Murphy, Polly V. Peers, Tom Manly

**Affiliations:** 1 University of Cambridge, UK; 2 University of Oxford, UK

**Keywords:** acquired brain injury, intolerance of uncertainty, mood disorder, neurological impairment, psychometrics, assessment

## Abstract

Intolerance of uncertainty (IU) is a risk factor for poor mental health. Acquired brain injury (ABI; for example, stroke, traumatic brain injury) often brings considerable uncertainty and increased mood disorder vulnerability. The Intolerance of Uncertainty Scale–Short Form (IUS-12) is a brief, well-validated IU measure in non-ABI samples, comprising two subscales, namely, Prospective Anxiety and Inhibitory Anxiety. Here, for the first time, we investigated its reliability and validity (*N* = 118), and factor structure (*N* = 176), in ABI. Both subscales had high test–retest reliability (intraclass correlation coefficients [ICCs] of .75 and .86) and were significantly associated with mood disorder symptoms. The two-factor model was superior to a one-factor IU model fit. Some fit statistics were less than optimal (standardized root mean square residual [SRMR] = 0.06, root mean square error of approximation [RMSEA] = 0.09); hence, exploration of other factor structures in other ABI samples may be warranted. Nonetheless, the IUS-12 appears suitable in ABI.

Acquired brain injuries (ABIs) such as from traumatic brain injuries (TBIs), stroke, encephalitis, and brain tumors are a significant public health concern, with costs of TBI and stroke alone being approximately £23.9 billion per year (Parsonage, 2016; Saka et al., 2009). Due to medical advances, more people survive ABIs and the number living with chronic disabilities has increased. While some will show minimal post-ABI impairments, others will require intensive, multidisciplinary neurorehabilitation to address physical, cognitive, and emotional changes (Turner-Stokes et al., 2016). Of particular relevance to the current study is the up to fivefold increased risk of mood disorder in people with ABI compared with the general population (Ayerbe et al., 2013; Fazel et al., 2014; Rapoport, 2012). Factors such as the severity of the negative consequences of the ABI are important contributors to this risk (Mitchell et al., 2017). However, there is also value in examining the influence of psychological risk factors that may have been present pre-ABI or emerge subsequently. Good reasons for doing so are that, as with the non-ABI population, identifying such vulnerabilities could inform the development of effective psychological interventions and identify those in most need of support.

One well-studied risk factor for mood disorder in the general population is intolerance of uncertainty (IU). IU has been defined as a “dispositional characteristic that reflects a set of negative beliefs about uncertainty and its implications” (Dugas & Robichaud, 2007, p. 24). In early theoretical models, IU was conceptualized as a stable trait unique to generalized anxiety disorder (GAD). When faced with uncertainty, it was argued that those with higher levels of IU would engage in pathological worry in an attempt to control it, in a way that was inadvertently likely to maintain/increase anxiety levels (Dugas & Robichaud, 2007). Similarly, it has been suggested that people with high IU tend to engage in a series of seemingly adaptive, but often actually counterproductive, strategies, including avoiding tasks with uncertain outcomes, delaying decision-making, seeking excessive information or reassurance, and overpredicting negative outcomes from ambiguous situations (Buhr & Dugas, 2009; Dugas & Robichaud, 2007; Koerner & Dugas, 2008; Sankar et al., 2017). While determining causal direction is challenging, it has been reported that priming participants to view uncertainty as negative was sufficient to induce temporary mood reductions and increased avoidance (Flores et al., 2018; Ladouceur et al., 2000; Mosca et al., 2016; San Martín et al., 2020). In intervention research, IU is considered a maladaptive schema within cognitive behavior therapy models in which negative evaluations about future uncertainty and avoidance of uncertainty are targeted in anxiety-reduction therapy (e.g., Dugas et al., 2022). Taken together, there is, therefore, evidence that IU is a *transdiagnostic* cognitive vulnerability (McEvoy et al., 2019; Rosser, 2019) associated with depression, generalized anxiety, and social anxiety (Boelen & Reijntjes, 2009; Carleton, 2016; Hong & Cheung, 2015; McEvoy et al., 2019; Shihata et al., 2016).

ABIs are life-threatening events that often cause considerable uncertainty about one’s health, abilities, occupation, and many other domains. “Coping with uncertainty” was a major theme in a meta-synthesis of nine qualitative studies of poststroke adjustment (Salter et al., 2008). Participants noted that their “*future became painfully and consciously uncertain*,” citing uncertainties over whether they might have another stroke, what had caused the stroke, irregular patterns of recovery, and unpredictable functional capabilities (Salter et al., 2008). Further qualitative research has proposed uncertainty as a “meta-theme” of adjustment post-ABI (Connolly et al., 2021; Cubis et al., 2018; Snell et al., 2020; Sørensen et al., 2020). Given these heightened levels of uncertainty and risk of mood disorder post-ABI, and the established links between IU and mental health, there has been surprisingly little quantitative work on IU in ABI. An important precursor to such work lies in identifying good measures. IU has been typically assessed in the general population using self-report questionnaires. It is possible that a reliable and valid measure of IU in the general population could be less effective in ABI, for example, due to problems comprehending items, poor memory for relevant experiences, or the much greater *levels* of uncertainty that some people face. Similarly, there may be a tendency to interpret items differently in the context of an ABI, for example, because a very unwelcome surprise event (the ABI) has occurred. Such issues have been examined in relation to other measures such as the Patient Health Questionnaire–9 (PHQ-9), in which an item such as “How often are you bothered by feeling tired or having little energy” may be strongly influenced by somatic consequences of an ABI. It is, therefore, necessary to test rather than assume the efficacy of measures developed for the general population in people with ABI (Law, 1987; Tate & Perdices, 2017). Such evaluation has already taken place for a range of self-report measures (e.g., PHQ-9: Fann et al., 2005; Generalized Anxiety Disorder–7 [GAD-7]: Teymoori et al., 2020) but has yet to be undertaken for IU. Specifically for *the Intolerance of Uncertainty Scale–Short Form*, this was the novel aim of the current study.

The original 27-item *Intolerance of Uncertainty Scale* (IUS; Buhr & Dugas, 2002), developed for GAD studies, was shortened to 12 items via exploratory factor analysis (*IU-Short Form*; IUS-12; Carleton et al., 2007). The IUS-12 has been argued to assess two latent factors, namely, Prospective Anxiety and Inhibitory Anxiety, reflecting orientation toward future uncertainty (“A small unforeseen event can spoil everything, even with the best of planning”) and avoidance responses to uncertainty (“When it’s time to act, uncertainty paralyses me”), respectively (Birrell et al., 2011). Previous research has demonstrated high levels of Prospective Anxiety lead to increases in pathological worry, thus is more strongly related to GAD (McEvoy & Mahoney, 2011; Shihata et al., 2016), whereas Inhibitory Anxiety generates avoidance and ruminative tendencies in depression (Saulnier et al., 2019) and paralysis in the face of peer evaluation in social anxiety disorder (Boelen & Reijntjes, 2009). The IUS-12 correlates strongly with the original 27-item version and scores have demonstrated good reliability and validity estimates outside of ABI, the latter being judged via relationships to measures of depression, generalized anxiety, social anxiety, and behavioral avoidance/reduced activity (McEvoy & Mahoney, 2011).

Given the psychometric strengths, and the practicality afforded by its brevity, the IUS-12 is, therefore, a strong candidate for evaluation in ABI. To this end, in the current study 118 people with ABI completed the IUS-12 together with measures of depression, anxiety, social anxiety, and behavioral avoidance/reduced activity. Subgroups of this initial sample repeated the IUS-12 four and 20 weeks later, when some of the mood measures were again administered. Thus, we assessed the concurrent validity, predictive validity, and test–retest reliability estimates of IUS-12 scores in this population in addition to undertaking some more exploratory analyses concerning IUS-12’s relationship to ABI severity and motivation measures.

A second question that we addressed was whether the patterns of covariance between IUS-12 items would be similar in an ABI sample to those reported in general population studies, and whether it seems to be capturing the same underlying constructs. As discussed, a two-factor structure (Prospective Anxiety and Inhibitory Anxiety) has been found to be a good fit for the IUS-12 and has been supported in several non-ABI samples (Birrell et al., 2011). The two-factor structure was thus used as the basis of our subscale reliability and validity investigations outlined above. In the second part of the article, we use confirmatory approaches to determine the fits of one-factor and two-factor structures of IUS-12 items to our ABI data set.

## Study Purpose

This study aims to evaluate the psychometric properties of the IUS-12 in adults with an ABI. We report on its test–retest reliability, concurrent and predictive validity, and internal consistency and factor structure.

## Method

We report how we determined our sample size, all data exclusions, all manipulations, and all measures in the study. This study received favorable opinion from the University of Cambridge Psychology Research Ethics Committee (PRE.2018.059). Participants were paid £6 per hour. The initial psychometric study was preregistered in August 2018 (https://osf.io/atbk3). The amended study that included the 20-week test–retest assessment was preregistered in July 2021 (https://osf.io/q7ktc). The data for all analyses are available on the Open Science Framework (https://osf.io/6c89x). All analyses were conducted using R statistical software version 4.1.2 (R Core Team, 2021).

### Study Design and Sample Size

Part 1 has a longitudinal observational design. Part 2 uses a cross-sectional observational design. The sample size for both parts was based on estimated numbers required to conduct a confirmatory factor analysis of the IUS-12. Based on the recommendations of MacCallum et al. (1999), the ideal sample size for a factor analysis should be based on study-specific participant to variable ratio and variable communality (i.e., proportion of variance attributable to a common underlying factor). For preestablished factors with high (*h* ^2^≥ .6) communality (see Hale et al., 2016),^1^ a minimum of 100 participants is sufficient. One-hundred twenty participants were recruited to allow for missing data and outliers.

### Participant Identification

Participants for both the factor structure and reliability and validity parts were recruited from four sources:

1. the Cambridge Cognitive Neuroscience Research Panel (CCNRP), a panel of adults with stroke and resected brain tumors confirmed via neuroradiological scans;
2. UK-based ABI charities, including the Stroke Association and Headway;
3. Social media ABI groups (e.g., via Twitter, Facebook);
4. Prolific Academic (hereafter “Prolific”), an online participant recruitment service (see https://prolific.co/).

Interested participants recruited via the Research Panel and ABI charities were invited to the MRC Cognition and Brain Sciences Unit to take part or were emailed a link to complete the study online. Participants recruited via social media responded to adverts asking for volunteers with ABI and participated online. With the onset of the first UK COVID-19 lockdown and resulting social distancing regulations, all subsequent participation was online.

On joining Prolific, individuals answer routine demographic questions, including whether they have sustained a “head injury.” Because responding “yes” could include a range of injuries, including those without neurological injury, we invited individuals recruited via Prolific to complete a prescreen survey to increase consistency with Research Panel and charity-recruited participants. The inclusion criteria for Prolific participants were the following:

1. Reported that the injury occurred at age 18 or older.
2. Reaffirmation of experiencing a head injury (i.e., confirming their initial endorsement at the point of joining Prolific) or self-report of another form of ABI such as stroke.
3. For those reporting a head injury, we applied an additional “rule-of-thumb” inclusion criterion of loss of consciousness of 30 min or longer. A relatively small group of participants (*n* = 16) gave responses indicating that they had sustained an *acquired brain injury* (i.e., not simply a “head injury”) but that loss of consciousness was either not of this duration or was unknown to them. These participants were included in the sample.
4. Having received treatment in A&E, hospitalization, and/or being admitted to a rehabilitation facility as a result of the brain injury.

## Part 1—Reliability and Validity of the IUS-12 in ABI

The aims of Part 1 analyses were to

1. estimate IUS-12 test–retest reliability in adults with ABI;
2. evaluate the concurrent and predictive validity of the IUS-12 against measures of depression, generalized anxiety, social anxiety, and activity/avoidance levels;
3. conduct exploratory analyses of the relationships between IUS-12 and motivation and ABI symptom severity.

Our preregistered hypotheses were as follows:

1. As in previous studies in adults without ABI, IUS-12 scores will demonstrate good test–retest reliability (greater than .70 intraclass correlation coefficient [ICC]).
2. The IUS-12 will be significantly positively predictive of self-reported generalized anxiety, social phobia, depression scales, and activity/avoidance scales completed at the same session and 20 weeks later (i.e., will show acceptable concurrent and predictive validity).

All other analyses were exploratory. The data for all analyses presented here are openly available on the Open Science Framework https://osf.io/6c89.

### Data Collection

At Time 1, participants completed a demographics form and a series of measures, including IUS-12, and of depression, generalized anxiety, social anxiety, activation/avoidance, motivation, and brain injury symptoms (see *Study Measures*). All participants were recontacted approximately 4 weeks later (range = 28–42 days, *M* = 31 days) to complete IUS-12 a second time (Time 2). Participants who completed Time 1 at any point after the onset of the first U.K. COVID-19 lockdown (March 23, 2020; potential *N* = 60) were also reinvited to a Time 3 data collection time point due to a separate research investigation on IU and COVID-19. Time 3 data collection took place roughly 20 weeks post-Time 1 (range = 140–154 days, *M* = 143 days).

### Data Cleaning Procedures

The following procedures were used for Part 1 and 2. Participants who took part from any source were excluded if they had incomplete data (defined as greater than 20% of data missing across all questionnaires). Online participants were additionally excluded if they met one or more of the following criteria:

1. Failing to pass all three embedded attention checks (e.g., instructed “Please select the response ‘sometimes’ for this item” within the questionnaire battery);
2. Completing the entire study session at a rate suggestive of incomplete attention (i.e., greater than 2.5 standard deviations below the mean).

The above resulted in the exclusion of *n* = 9 participants, with *n* = 7 excluded due to fast completion time and *n* = 2 excluded due to failing to pass all three attention checks.

For Part 1 specifically, to account for missingness at the item level, total scores for each measure were aggregated into an estimated total score using the mean from available items. If a participant had more than 20% of items missing on subscales or scale totals, these cases were also excluded.

### Study Measures

The *Intolerance of Uncertainty Scale–Short Form* (IUS-12; Carleton et al., 2007) is a 12-item trait measure of responses to uncertainty, ambiguous situations, and the future. The IUS-12 has two subscales: Prospective Anxiety and Inhibitory Anxiety. Items are rated on a 5-point Likert-type scale ranging from 1 (*not at all characteristic of me*) to 5 (*entirely characteristic of me*). Prospective Anxiety scores range from 7 to 35, and Inhibitory Anxiety scores range from 5 to 25. Higher scores on the subscales indicate greater difficulties with Prospective and/or Inhibitory Anxiety. A total IU score can also be calculated.

The *European Brain Injury Questionnaire–Self* (EBIQ-S; Teasdale et al., 1997) is a 63-item measure developed to assess the subjective experience of brain injury and its associated symptoms. Participants rate the frequency of each symptom in the past month on a 3-point Likert-type scale (1 = *not at all*, 2 = *a little*, and 3 = *a lot*). The EBIQ-S can be used to assess overall ABI symptom frequency, with possible scores ranging from 63 to 189, and/or subscale scores can be calculated. Although the original EBIQ-S had some subscales with poor internal consistency (Teasdale et al., 1997), in the present study Cronbach’s alpha for the subscales ranged from acceptable to good (αs = .64–.89); therefore, these subscales were subsequently used in analysis. These subscales represent common functional categories in ABI (*Somatic, Cognitive, Impulsivity, Depression, Physical, Isolation, Motivation, and Communication*).

The *Patient Health Questionnaire–8* (PHQ-8; Kroenke et al., 2009) is a measure of depression. The scale contains eight items assessing the frequency of experiencing depression symptoms in the past 2 weeks (e.g., “Little interest or pleasure in doing things”), with ratings including 0 (*not at all*), 1 (*several days*), 2 (*more than half the days*), and 3 (*nearly every day*). A final item asks participants to rate the extent to which any endorsed symptoms impeded daily functioning, with options *not at all difficult*, *somewhat difficult*, *very difficult*, and *extremely difficult* scored from 0 to 3. Total scores on the PHQ-8 range from 0 to 24, with the functioning item scored separately. Save for a single missing item on suicidal ideation, the PHQ-8 is identical to the PHQ-9 that has good internal consistency and test–retest reliability in adults with and without ABI (Fann et al., 2005; Kroenke et al., 2010; von Steinbuechel et al., 2021). A cut score of at least five symptoms, including one cardinal symptom, has the best sensitivity and specificity in adults with TBI (Fann et al., 2005). The PHQ-8 was used here in preference to the PHQ-9 due to institutional safeguarding policies prohibiting questions about suicide for anonymous online participants (to whom immediate support could not be offered).

The GAD-7 (Spitzer et al., 2006) scale has seven items assessing symptoms in the past 2 weeks (e.g., “Worrying too much about different things”). An additional item asks participants to rate the extent to which any endorsed symptoms made daily functioning difficult. The symptomatology items are rated from 0 to 3, with options including *not at all*, *several days*, *more than half the days*, and *nearly every day*. The item assessing function is rated from 0 to 3, with options including *not at all difficult*, *somewhat difficult*, *very difficult*, and *extremely difficult*. Total scores range from 0 to 21, with the functioning item scored separately. The GAD-7 has demonstrated excellent internal consistency, split-half reliability, and good test–retest reliability in adults with ABI (Teymoori et al., 2020; von Steinbuechel et al., 2021), with cut scores of 10 representing optimal sensitivity and specificity (Kroenke et al., 2010).

The *Social Phobia Inventory* (SPIN; Connor et al., 2000) is a 17-item measure of social anxiety. The SPIN consists of subscales assessing fear (e.g., “Parties and social events scare me”), avoidance (e.g., “I avoid activities in which I am the center of attention”), and physiological symptoms (e.g., “Heart palpitations bother me when I am around people”) experienced in the past week. Items are rated on a 5-point Likert-type scale, ranging from 0 (*not at all*) to 4 (*extremely*), with total scores ranging from 0 to 68. The SPIN demonstrates good internal consistency, convergent validity, and test–retest reliability in adults with and without ABI (Connor et al., 2000; Curvis et al., 2018; Jimenez-Zambrano et al., 2020). A cut score of 19 has good sensitivity and specificity in distinguishing social anxiety (Connor et al., 2000).

The *Behavioral Activation for Depression Scale–Short Form* (BADS-SF; Kanter et al., 2007, 2009) is a nine-item, shortened version of the original 25-item BADS. The scale measures avoidance and activity levels considered central to depression. Participants are instructed to respond based on the previous week. The BADS-SF consists of two subscales: Activation (e.g., “I engaged in many different activities”), and Avoidance/Rumination (e.g., “I spent a long time thinking over and over about my problems”). Items are rated on a 7-point Likert-type scale, ranging from 0 (*not at all*) to 6 (*completely*). Possible Activation scores range from 0 to 36, with higher scores indicating better activation. Possible Avoidance/Rumination scores range from 0 to 18, with higher scores indicating heightened avoidance and rumination. The BADS has good factor structure, internal consistency, test–retest reliability, and construct validity in adults with and without ABI (Grenawalt et al., 2022; Kanter et al., 2007, 2009).

*Brain Injury Rehabilitation Trust Motivation Questionnaire–Self* (BMQ-S; Oddy et al., 2008) is a 34-item questionnaire designed to measure difficulties with motivation following ABI. Items are rated on a 4-point Likert-type scale, with the response options including *always*, *often*, *sometimes*, and *never*. Total scores range from 34 to 136, with higher scores indicating greater *problems* with motivation (i.e., not motivated). Examples of items include “It’s hard to get started, even when I know I’ve got something to do” and “I have lots of get up and go” (reverse coded). The BMQ-S has good internal consistency, test–retest reliability and high split-half reliability in ABI (Kusec et al., 2018; Oddy et al., 2008).

### Statistical Analyses

The results were, first, analyzed irrespective of the phase of the COVID-19 pandemic during data collection. A Shrout & Fleiss (2,1) ICC was used to estimate test–retest reliability for IUS-12 subscales across timepoints (Shrout & Fleiss, 1979). ICC estimates were obtained using the *psych* R package (Revelle, 2021). Both complete case analysis and maximum likelihood estimation for missing cases were conducted. Cichetti’s (1994) guidelines for evaluating good clinical significance (ICC ≥ .60) were used to guide interpretation of the ICC. To determine the standard error of measurement (SEM) of the IUS-12, the following formula was used:

$$\mathrm{SEM}=\sigma \times \sqrt{1-r}$$

where σ is the standard deviation of the IUS-12 and *r* is its reliability. The standard deviation of the IUS-12 per subscale at Time 1 was used to calculate the SEM. Spearman’s *rho* correlations were estimated to examine the rank-order stability of IUS-12 scores across time points to detect whether individuals scoring within a certain rank at Time 1 continued to do so at Time 2 and Time 3 despite any time-related factors (e.g., pandemic).

Correlation matrices of IU subscales to depression, generalized anxiety, social anxiety, and activity levels/avoidance (PHQ-8, GAD-7, SPIN, and BADS-SF) scores were estimated to assess concurrent validity. Bonferroni-adjustments using the *psych* R package (Revelle, 2021) corr.test function were conducted across all correlations. Multivariable linear regressions were conducted to assess whether IUS-12 subscales uniquely related to depression, generalized anxiety, and social anxiety when considered in the same model. To estimate predictive validity, IUS-12 scores at Time 1 were used to predict depression, generalized anxiety, social anxiety, and activity levels/avoidance scores at Time 3. We examined the effect of removing influential cases (as defined by a Cook’s distance of greater than 4 divided by the sample size; Cook, 1977). Inclusion or exclusion of influential cases made little to no difference in analyses. Where differences were observed in regression models, these are reported in text.

Exploratory analyses were conducted on the relationships between IUS-12 scores and BMQ-S motivation scores, and whether IUS-12 subscale scores contributed unique variance to PHQ-8, GAD-7, and SPIN scores above ABI symptoms as reported on the EBIQ-S using Time 1 data.

## Part 1 Results

### Demographics

A total of 118 participants participated at Time 1 (demographics shown in Table 1), with 82 participants taking part at Time 2. Finally, 48 participants (whose Time 1 data were collected during COVID-19) completed Time 3. A detailed account of missingness due to attrition and its relationship to study variables is presented in the *Supplementary Materials.* In brief, there were no differences in IUS-12 and other measures between those retained and those who withdrew except for EBIQ-Impulsivity and EBIQ-Physical scores.

**Table 1.** Participant Characteristics With Data Collected at Time 1.

Participants (*N* = 118)	Value	Min–max
Gender, *n* (%)		
Male	62 (52.50)	
Female	56 (47.50)	
Age, *M* (*SD*)	48.07 (14.17)	24–78
Ethnicity, *n* (%)		
White British	108 (91.50)	
Mixed ethnicity	5 (4.20)	
White Other	3 (2.50)	
Black British	2 (1.80)	
Highest level of education, *n* (%)		
GCSE/O-level	15 (12.70)	
A-levels	17 (14.40)	
Diploma	21 (17.80)	
Bachelor’s degree	34 (28.80)	
Postgraduate degree	23 (19.50)	
Other	8 (6.80)	
Living situation, *n* (%)		
Living with partner/spouse	57 (48.30)	
Living with family	37 (31.40)	
Living alone	24 (20.30)	
Work status, *n* (%)		
Full time	52 (44.10)	
Part time	18 (15.30)	
Retired (including medical retirement)	22 (18.60)	
Unemployed	15 (12.70)	
Other	11 (9.30)	
Type of ABI, *n* (%)		
Traumatic brain injury	66 (55.90)	
Stroke	23 (19.50)	
Tumor	25 (21.20)	
Other	4 (3.40)	
Years post-ABI, *M* (*SD*)	11.01 (8.73)	0.20–40.80
Reported duration of loss of consciousness in minutes, median^a^	30	0–20,160
Recruitment source, *n* (%)		
Prolific	57 (48.30)	
Research panel	33 (28.00)	
Social media	23 (19.50)	
ABI charity	5 (4.20)	
Method of assessment, *n* (%)		
Online	96 (81.40)	
In person	22 (18.60)	

*Note.* Of note, 58 participants (49.2% of the sample) completed the study prior to the first COVID lockdown in the United Kingdom (before March 2020) and the remaining 60 participants (50.8%) during COVID (post March 2020). GCSE = General Certificate of Secondary Education; ABI = acquired brain injury.

a Only collected for individuals reporting a “head injury” or “traumatic brain injury.”

### Test–Retest Reliability

Mean IUS-12 Prospective Anxiety scores did not differ between time points (*F*_2, 245_ = 0.91, *p* = .41), nor did mean IUS-12 Inhibitory Anxiety scores (*F*_2, 245_ = 0.54, *p* = .59). For the Prospective Anxiety subscale, rank-order stability was high from Time 1 to Time 2 (ρ = 0.57), Time 1 to Time 3 (ρ*=* 0.72), and Time 2 to Time 3 (ρ = 0.71). For the Inhibitory Anxiety subscale, rank-order stability was high from Time 1 to Time 2 (ρ = 0.74), Time 1 to Time 3 (ρ*=* 0.74), and Time 2 to Time 3 (ρ = 0.81).

In general, ICCs using data from all time points were excellent (ICCs ≥ .75). A summary of ICC estimates is presented in Table 2. The SEM for the IUS-12 Prospective Anxiety subscale was 2.84 using Time 1 and Time 2 data, and 2.20 using data from Times 1 to 3. For the IUS-12 Inhibitory Anxiety subscale, the SEM was 1.94 for Time 1 to Time 2 data, and 1.47 for Times 1 to 3 data. In summary, whether the absolute scores are considered, or individuals’ rank order, the IUS-12 showed excellent test–retest reliability in participants with ABI.

**Table 2.** Average 2,1 Intraclass Correlation Coefficients With 95% Confidence Intervals for IUS-12 Subscale Scores.

IUS-12 subscale	Complete cases Time 1 to Time 2 (*N* = 82)	Complete cases Times 1, 2, and 3 (*N* = 48)	Full sample Times 1, 2, and 3 (*N* = 118)
Prospective anxiety	.75 [.61, .84]	.85 [.76, .91]	.85 [.80, .89]
Inhibitory anxiety	.86 [.79, .91]	.92 [.87, .95]	.91 [.87, .93]

*Note.* The first column provides estimates for complete cases at Time 1 to Time 2, with the second column estimates for complete cases at Times 1, 2, and 3. The third column provides an estimate of the ICC given the missing data using maximum likelihood estimation. IUS-12 = Intolerance of Uncertainty Scale–12.

### Concurrent Validity

**Figure 1.**
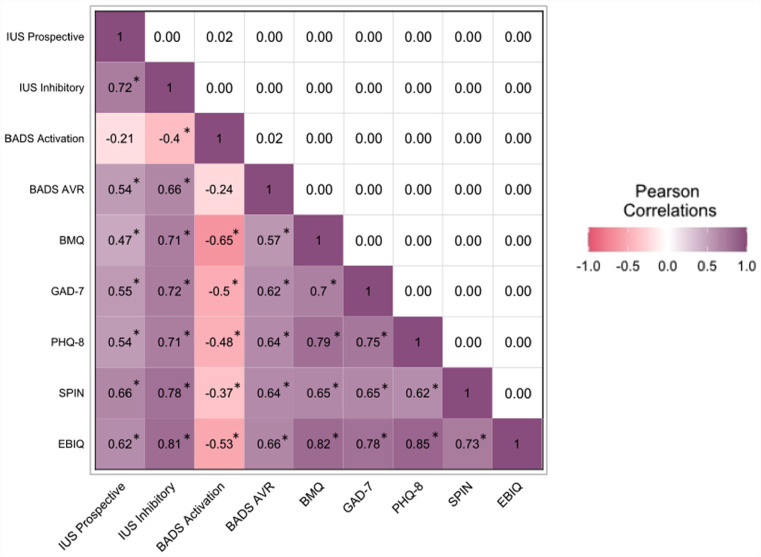
Bonferroni-Adjusted Correlation Matrix of Study Variables at Time 1 (N = 118). *Note*. All 36 comparisons were alpha-adjusted. Adjusted *p* values are presented along the upper diagonal, while correlation values are shown along the lower diagonal. IUS = Intolerance of Uncertainty Scale–Short Form; BADS = Behavioral Activation for Depression Scale; BADS AVR = BADS Lack of Avoidance/Rumination; BMQ = Brain Injury Rehabilitation Trust Motivation Questionnaire; GAD-7 = Generalized Anxiety Disorder–7; PHQ-8 = Patient Health Questionnaire–8; SPIN = Social Phobia Inventory; EBIQ = European Brain Injury Questionnaire. *p < .001 after alpha adjustment.

A correlation matrix of Time 1 variables is presented in Figure 1. Probabilities for all correlations were alpha-adjusted using a Bonferroni procedure. As displayed, Prospective Anxiety and Inhibitory Anxiety were significantly correlated with depression scores, generalized anxiety scores, social anxiety, and BADS-Avoidance scores though not BADS-Activation scores following alpha adjustment. Hypotheses regarding concurrent validity of IUS-12 were therefore confirmed, with the exception of BADS-Activation scores. In exploratory analyses, both Prospective Anxiety and Inhibitory Anxiety were significantly correlated with worse BMQ-S motivation and greater ABI symptom severity.

In general, Inhibitory Anxiety correlated more strongly with study measures compared with Prospective Anxiety.^2^ Visualizations of the distributions of IUS-12 subscales at Time 1 per clinical cut-off scores are shown in *Supplementary Figure S1*.

### Predictive Validity

Prospective Anxiety at Time 1 was correlated with Time 3 depression (PHQ-8; *r* = .60, *p* < .001), generalized anxiety (GAD-7; *r* = .58, *p* < .001), social anxiety (SPIN; *r* = .51, *p* < .001), avoidance (BADS-Avoidance; *r* = .45, *p* = .00121), and activity levels (BADS-Activation; *r* = −.35, *p* = .0148). Inhibitory Anxiety showed a similar pattern, but was overall more strongly correlated with mood outcomes (correlations with PHQ-8: *r* = .67, *p* < .001; GAD-7: *r* = .68, *p* < .001; SPIN: *r* = .71, *p* < .001; BADS-Avoidance: *r* = .55, *p* < .001; BADS-Activation: *r* = −.35, *p =* .00114). The predictive validity hypotheses of IUS-12 scores for mood measures were thus confirmed.

### Exploratory Analysis—Relationship of IU to Poor Mental Health After Controlling for ABI Symptoms

As shown in Figure 1, Prospective and Inhibitory Anxiety were significantly positively correlated with ABI symptoms as measured by the EBIQ-S; the greater the impact of the ABI across various domains of function, the higher the IUS-12 subscale scores tended to be. As discussed, the consequences of ABI symptoms (e.g., mobility issues or cognitive problems) on everyday life are a risk factor for post-ABI mood disorder (Juengst et al., 2017; Shi et al., 2017). To determine whether IUS-12 scores explained unique variance in depression, generalized anxiety, and social phobia symptom scores *above* ABI symptoms, three cross-sectional multivariable regressions were conducted predicting PHQ-8, GAD-7, and SPIN scores. Each regression model was evaluated for influential cases. In the case of the GAD-7 and SPIN models, inclusion or exclusion of influential cases made no difference to the predictive value of Predictive and Inhibitory Anxiety. Influential case removal in the PHQ-8 model resulted in Inhibitory Anxiety becoming a statistically significant predictor; however, this was not considered to be practically meaningful given that all other regression assumptions were met and that in ABI samples, an array of more extreme scores are common within data sets. Therefore, to avoid misconstruing the data, here we present all regression results inclusive of highly influential cases.

The EBIQ-S subscales were entered into a multivariable regression model with Prospective Anxiety and Inhibitory Anxiety scores. The Depression subscale of the EBIQ-S was not included due to its obvious conceptual overlap with mood measures. An overview of regression results is in Table 3.

**Table 3.** Full Regression With Standardized Coefficients (β) and Standard Errors Using Complete Case Analysis (N = 118).

Dependent variable	PHQ-8		GAD-7		SPIN	
	β (*SE*)	*p*	β (*SE*)	*p*	β (*SE*)	*p*
EBIQ Predictors						
Physical	–0.07 (0.24)	.41	–0.19 (0.24)	.04*	0.33 (0.67)	<.001***
Somatic	0.19 (0.14)	.011*	0.35 (0.14)	<.001***	–0.04 (0.39)	.62
Cognitive	0.19 (0.12)	.07	0.03 (0.12)	.79	0.00 (0.32)	.99
Motivation	0.41 (0.22)	<.001***	0.05 (0.22)	.63	–0.04 (0.62)	.69
Impulsivity	0.15 (0.12)	.08	0.27 (0.13)	.009**	–0.22 (0.35)	.03*
Isolation	0.01 (0.27)	.95	0.06 (0.31)	.55	0.15 (0.76)	.10
Communication	–0.07 (0.30)	.53	0.14 (0.13)	.22	0.13 (0.85)	.26
Intolerance of uncertainty						
Inhibitory anxiety	0.15 (0.12)	.12	0.33 (0.13)	.002**	0.39 (0.35)	<.001***
Prospective anxiety	–0.01 (0.09)	.87	–0.10 (0.10)	.26	0.28 (0.26)	.002**

*Note.* Brain injury symptoms as measured via the EBIQ-S. Bolded text indicates that a predictor signficantly explained variance within each model. PHQ-8 = Patient Health Questionnaire-8; GAD-7 = Generalized Anxiety Disorder-7; SPIN = Social Phobia Inventory; EBIQ = European Brain Injury Questionnaire; EBIQ-S = EBIQ-Self.

* *p* < .05. **p < .01. ***p < .001.

In the depression model, only EBIQ-Motivation scores (β = 0.41, *SE* = 0.22) and EBIQ-Somatic scores (β = 0.19, *SE* = 0.14) showed statistically unique associations with PHQ-8 scores, with the full model accounting for 71% of the variance (*F*_9, 105_ = 32.63, *p* < .001). Prospective and Inhibitory Anxiety did not, therefore, predict PHQ-8 scores once ABI symptoms were considered.

The full GAD-7 generalized anxiety model accounted for 66% of the variance (*F*_9, 103_ = 24.99, *p* < .001). EBIQ-Somatic scores had the strongest contribution (β = 0.35, *SE* = 0.14), followed by Inhibitory Anxiety (β = 0.33, *SE* = 0.13), EBIQ-Impulsivity (β = 0.27, *SE* = 0.13), and EBIQ-Physical (β = −0.19, *SE* = 0.24) scores.

In the social anxiety model, Inhibitory Anxiety scores had the greatest contribution (β = 0.39, *SE* = 0.35), followed by EBIQ-Physical (β = 0.33, *SE* = 0.67), IUS-12 Prospective Anxiety (β = 0.28, *SE* = 0.26), and EBIQ-Impulsivity (β = −0.22, *SE* = 0.35) scores, capturing 67% of the variance (*F*_9, 105_ = 26.91, *p* < .001).

Taken together, the multivariable regression results demonstrate that Inhibitory Anxiety and Prospective Anxiety robustly explain unique variance in generalized anxiety and social anxiety above ABI symptoms; however, they do not seem to reliably predict depression severity in ABI.

## Part 2—Internal Consistency and Factor Structure of the IUS-12

Part 2 aimed to evaluate the internal consistency and factor structure of the IUS-12 in adults with an ABI. For Part 2 only, IUS-12 data collected from the participants recruited as above were combined with data from another study that we had conducted in which participants with ABI had also completed the IUS-12 (Kusec et al., 2020). This was done to prevent model nonconvergence (MacCallum et al., 1999) in an ABI sample.

### Data Collection

After completing a basic demographics form, participants completed the IUS-12 as part of a larger battery of questionnaires used to evaluate IUS-12 validity as in Part 1 above, or as part of the separate research study (Kusec et al., 2020).

### Measures

The *Intolerance of Uncertainty Scale–Short Form* (IUS-12; Carleton et al., 2007) is a 12-item trait measure of responses to uncertainty, ambiguous situations, and the future (see Part 1 for details).

### Data Cleaning Procedures

These were as described in Part 1 except procedures stated as specific to Part 2.

### Statistical Analyses

To determine the internal consistency of the IUS-12, Cronbach’s alpha was used and evaluated using Bland and Altman’s (1997) acceptability criteria (α > .70). To estimate the factor structure of the IUS-12, two confirmatory factor analyses were estimated using the *lavaan* package for R (Rosseel, 2012). Although previous evidence suggests that a two-factor structure is superior to a one-factor structure in the general population (Carleton et al., 2007), the scale has not been used in ABI. Hence, both one-factor and two-factor models were estimated. A robust diagonally weighted least squares (WLSMV) estimator was used, and items were treated as ordinal data. Cases with missing items on the IUS-12 were removed using listwise deletion. Fit indices used to evaluate the models included

1. Chi-square (χ^2^) statistic (lower and nonsignificant test statistic desirable),
2. Comparative fit index (CFI) and Tucker–Lewis index (TLI) (values greater than 0.95 indicate acceptable fit; Schermelleh-Engel et al., 2003),
3. The root mean square error of approximation (RMSEA) (values lower than 0.08 indicate acceptable fit; Schermelleh-Engel et al., 2003), and
4. The standardized root mean square residual (SRMR) (values lower than 0.05 indicate good fit; Schermelleh-Engel et al., 2003).

To formally compare models, chi-square difference between models was used, with lower values indicating better fit (Schermelleh-Engel et al., 2003).

## Part 2 Results

### Demographics

A total of 176 participants were included in analyses (see Table 4). Participants were generally in the chronic stages of ABI, and nearly half had experienced a TBI. There was an equal balance in gender, though White British participants were overrepresented relative to UK population norms.

**Table 4.** Study 2 Participant Characteristics.

Participants (*N* = 176)	Value	Min–max
Gender, *n* (%)		
Male	92 (52.30)	
Female	84 (47.70)	
Age, *M* (*SD*)	49.81 (13.63)	24–78
Ethnicity, *n* (%)		
White British	161 (91.50)	
Mixed Ethnicity	5 (2.80)	
White Other	5 (2.80)	
Black British	4 (2.30)	
Asian British	1 (0.60)	
Highest level of education, *n* (%)		
GCSE/O-level	24 (13.60)	
A-levels	29 (16.50)	
Diploma	21 (11.90)	
Bachelor’s degree	54 (30.70)	
Postgraduate degree	36 (20.50)	
Other	12 (6.80)	
Living situation, *n* (%)		
Living with partner/spouse	78 (44.30)	
Living with family	57 (32.40)	
Living alone	40 (22.70)	
Other	1 (0.60)	
Employment status, *n* (%)		
Full time	55 (31.20)	
Part time	24 (13.60)	
Retired (including medical retirement)	48 (27.30)	
Unemployed	29 (16.50)	
Other	20 (11.40)	
Type of ABI, *n* (%)		
Traumatic brain injury	87 (49.40)	
Stroke	50 (28.40)	
Tumor	29 (16.50)	
Other	10 (5.70)	
Years post-ABI, *M* (*SD*)	9.77 (8.72)	0.20–41
Reported duration of loss of consciousness in minutes, median (*n =* 87)^a^	30	0–64,080
Recruitment source, *n* (%)		
Prolific	57 (32.40)	
Research panel	41 (23.30)	
Social media	36 (20.50)	
ABI charity	24 (13.60)	
NHS ABI services	18 (10.20)	
Method of assessment—*n* (%)		
Online	130 (73.90)	
In person	46 (26.10)	

*Note.* Of note, 82 participants (46.6%) completed the study prior to the first UK COVID-19 lockdown and the remaining 94 participants (53.4%) during COVID-19. GCSE = General Certificate of Secondary Education; ABI = acquired brain injury.

a Only collected for individuals reporting a “head injury” or “traumatic brain injury.”

### IUS-12 Scale Summary

The IUS-12 was, overall, feasible to use with adults with ABI. Across all participants, very few items were left blank (Item 2 omitted by two participants, and Items 3, 4, 9, and 12 omitted by one participant each). All items elicited a full range of responses on the 5-point Likert-type scale. An overview of IUS-12 scale statistics is shown in *Supplementary Table 1*.

The average score on the IUS-12 total for participants was 34.57 (*SD =* 10.22). IUS-12 scores were overall somewhat elevated relative to previous studies with undergraduate students and the general population pre-COVID (*M* = 25.85, *SD* = 9.45; Carleton et al., 2007), although lower than those seeking treatment for psychological difficulties (*M* = 37.49, *SD* = 11.40; Carleton et al., 2007). Mean scores in the current study were comparable with general population scores during COVID-19 (*M* = 34.24, *SD* = 11.07; Bavolar et al., 2023).

IUS-12 scores did not differ according to gender (*t* = −0.92, *p* = .36), living situation (*F*_3,172_ = 0.26, *p* = .85), work status (*F*_5,170_ = 1.07, *p =* .38), or level of education (*F*_7,168_ = 0.95, *p* = .47). Interestingly, IUS-12 scores additionally did not differ with respect to whether data were collected pre- or during COVID-19 (*t* = 0.13, *p =* .89), whether data were collected in person or online (*t* = −0.88, *p =* .38), or by type of ABI (*F*_3,172_ = 2.07, *p* = .11). When evaluating whether there were differences in IUS-12 scores across recruitment sources (*F*_4,171_ = 3.64, *p* = .01), only Research Panel participants had significantly lower IUS-12 scores than those recruited via social media (*t* = 7.60, *p* < .01); otherwise, there were no differences on IUS-12 scores across recruitment sources. IUS-12 scores additionally did not differ between participants recruited specifically for this study and participants recruited from the CCNRP (*t* = −1.57, *p =* .12). IUS-12 scores did not correlate with self-reported duration of loss of consciousness following ABI (*r* = .05, *p* = .70) but were correlated with lower age (*r* = −.31, *p* < .001). The 16 participants who reported an ABI but whose duration of loss of consciousness was less than 30 min or was unknown did not differ from the rest of the sample on IUS-12 scores (*t* = −0.99, *p* = .33).

### Internal Consistency

Internal consistency estimates of the IUS-12 scale total were excellent (α = .90, 95% confidence interval [CI] = [.88, .92]). The Prospective Anxiety subscale (α = .81, 95% CI = [.76, .85]) and Inhibitory Anxiety subscale (α = .88, 95% CI = [.85, .91]) also demonstrated good and excellent internal consistency estimates, respectively.

### Factor Structure

**Figure 2.**
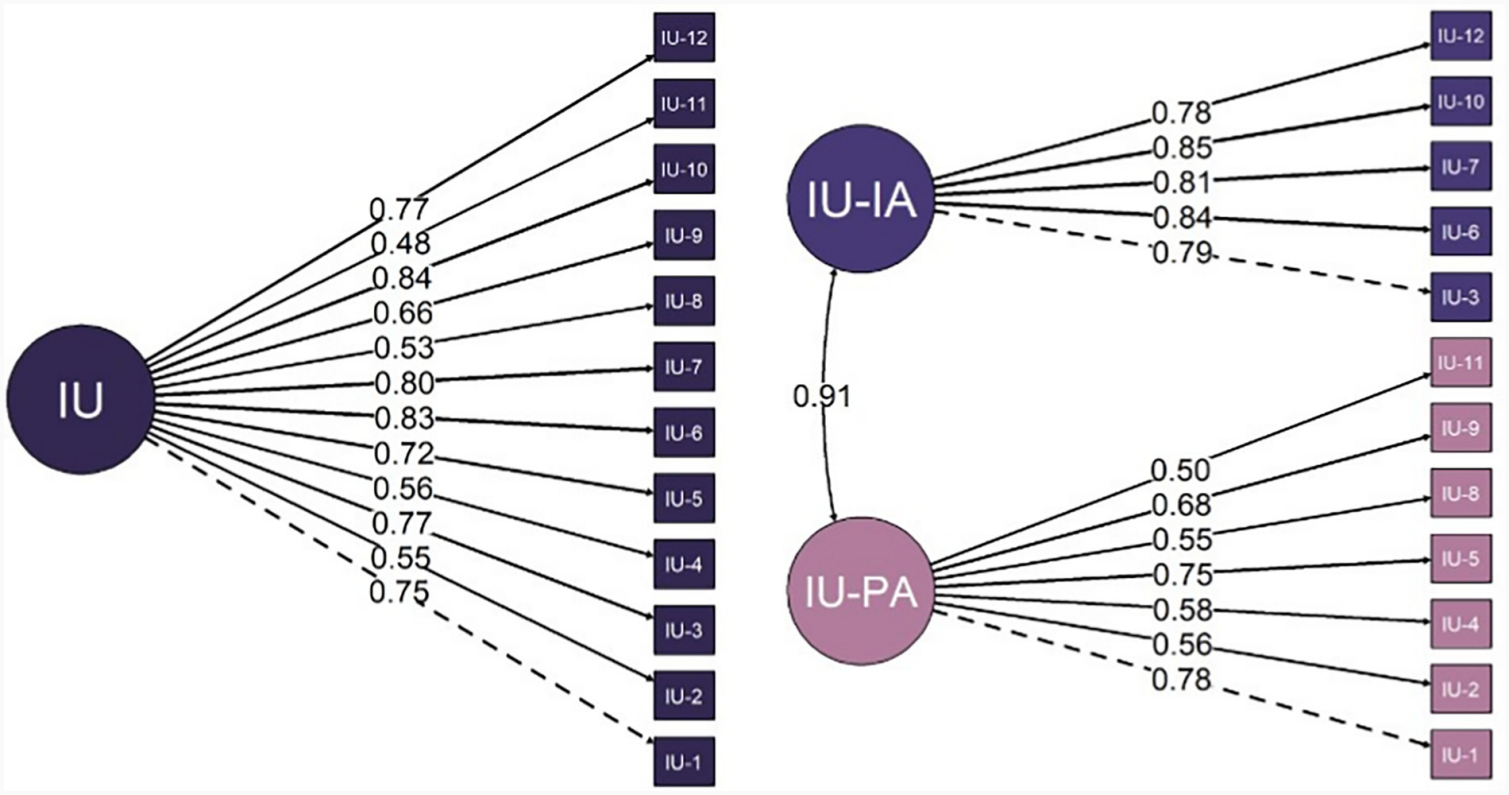
Confirmatory Models of a One-Factor IUS-12 Solution (Left) and the Original Prospective and Inhibitory Two-Factor IUS-12 (Right). *Note*. Numbers on each line indicate the standardized factor loadings (λ). Dashed lines indicate the first item that was fixed to the group factor. Covariance is expressed between the latent factors. IUS-12 = Intolerance of Uncertainty Scale–12;IU = intolerance of uncertainty; IU-IA = IU-Inhibitory Anxiety; IU-PA = IU-Prospective Anxiety.

A comparison of the one-factor and two-factor model is shown in Figure 2. First, the fit of a one-factor model of the IUS-12 was estimated. This displayed poor to acceptable fit statistics: χ^2^ = 143.51, *p* < .001, CFI = 0.96, TLI = 0.95, SRMR = 0.07, RMSEA = 0.10 (90% CI = [0.08, 0.12]), Scaling Correction Factor = .80. Next, the fit of a two-factor model, with items sorted to represent the previously established subscales Prospective Anxiety and Inhibitory Anxiety, was estimated. This displayed improved fit statistics, χ^2^ = 129.44, *p* < .001, CFI = 0.97, TLI = 0.96, SRMR = 0.06, RMSEA = 0.09 (90% CI = [0.07, 0.11]), Scaling Correction Factor = .78, and was a significant improvement from the one-factor model (Δχ^2^= 11.55, Δ*df* = 1, *p* < .001). Despite the improved fit, from a conservative perspective, the fit of the previously established two-factor model remained less than ideal, specifically for the SRMR and RMSEA estimates (Schermelleh-Engel et al., 2003). However, SRMR and RMSEA estimates can be positively biased in smaller samples and result in unduly rejecting admissible models (Hu & Bentler, 1999).^3^ Due to the otherwise acceptable fit of the two-factor model here, and the extensive literature supporting a two-factor structure (Birrell et al., 2011), we propose that a two-factor IUS-12 is additionally suitable in ABI.

## Discussion

The purpose of this study was to evaluate the reliability, validity, and factor structure of the Intolerance of Uncertainty Scale–Short Form (IUS-12; Carleton et al., 2007) in people with ABI.

### IUS-12 Reliability in ABI

In non-ABI populations, the IUS-12 has shown strong test–retest reliability estimates for its total score over 2 weeks (*r* = .77; Khawaja & Yu, 2010) and over 12 weeks (ICC = .87; Wilson et al., 2020). The results here additionally showed good reliability estimates for both subscales in ABI across 4 weeks (Prospective Anxiety ICC = .75; Inhibitory Anxiety ICC = .86) and 20 weeks (ICC = .85 and .92) when using complete cases. This test of IUS-12 stability is a valuable addition to not only the ABI literature, but to conceptualizations of IU as a relatively stable trait. In terms of reliability, therefore, the results support the utility of the IUS-12 in chronic ABI. Participants here were on average 11 years post-ABI (range = 3 months–40 years), and it is worth noting that IUS-12 scores could be more volatile during the first year following an ABI, a period often characterized by instabilities and the onset of mental health difficulties (Gould et al., 2011; Ouellet et al., 2018). Determining whether this volatility in earlier stages of ABI affects responses requires further study. It is also important to acknowledge that study attrition may have affected ICC estimates. Although we find no evidence that participants who withdrew differed on Time 1 IUS-12 scores (see *Supplementary Materials*), test–retest reliability estimates over 20 weeks in particular may be inflated due to smaller sample sizes, as this can result in higher homogeneity within participants (Bates et al., 1996). In addition, there was less than optimal power to detect a minimum ICC of .60 against a null ICC of .30 in 48 participants (power = 74.8%).

### IUS-12 Concurrent and Predictive Validity in ABI

Previous studies in non-ABI populations have shown associations between higher IU and increased depression, anxiety social anxiety, behavioral avoidance, and reduced activity (Carleton, 2016; Flores et al., 2018; Hong & Cheung, 2015; McEvoy et al., 2019; Mahoney & McEvoy, 2012; Sankar et al., 2017; Shihata et al., 2016; Wake et al., 2021). The results here show a highly consistent pattern, with strong correlations between Prospective Anxiety and Inhibitory Anxiety scores and depression, generalized anxiety, social anxiety, avoidance, and reduced activity administered concurrently. When examined longitudinally, Inhibitory Anxiety demonstrated stronger correlations with *all* measures. It is possible that stronger predictive validity estimates of Inhibitory Anxiety items for mood, activity, and avoidance measures are a feature of the factor structure of the IUS-12 in our sample. As discussed in relation to Part 2, the factor loadings for Prospective Anxiety were lower relative to Inhibitory Anxiety loadings, which could moderate respective relationships to mental health measures.

In exploratory analyses, we found that higher IU was associated with lower motivation. Given the conceptual relationship among activity, avoidance, and low motivation, this result is not surprising. Detecting heightened IU may be one route to identifying a cluster of likely mood and behavioral/cognitive characteristics worthy of follow-up. Whether these IU-motivation links are observed outside of ABI is an interesting question for further study. Possibly, ABI has unique physical and cognitive impairment–related motivational barriers that influenced the observed patterns here.

### IU, ABI Symptoms, and Relationship to Mood Measures

In an exploratory analysis, both IUS-12 subscale scores were positively correlated with self-reported ABI symptoms on the European Brain Injury Questionnaire (EBIQ). Several factors may explain this. First, the life-threatening experience of the ABI may foster a more negative attitude toward uncertainty (i.e., unexpected events with very negative outcomes *do* occur). Second, knowledge of one’s own acquired difficulties (e.g., slowed thinking, memory or language problems, perceptual or movement impairments) could lead to greater wariness and avoidance of uncertain situations (Salter et al., 2008). Third, IU has been posited as a risk factor for subsequent low mood and anxiety (McEvoy et al., 2019). Pre-ABI high IU may, therefore, exacerbate negative responses to the ABI and elevate the impact of mood symptoms. Finally, post-ABI mood disorder, which is itself related to symptom severity, may potentiate higher IU. All or some of these factors may combine in complex ways to produce the observed patterns. We only find a contribution of Inhibitory Anxiety to generalized anxiety (β = 0.33) and social anxiety (β = 0.39). It is possible that difficulties faced by some people with ABI are a sufficient explanation for low mood, and that an additional effect of IU was not easy to detect when taking these into account. Prospective Anxiety, being linked to social anxiety only, may be a useful marker differentiating social anxiety versus other forms of anxiety in ABI. Inhibitory Anxiety by contrast may prove to be a valuable transdiagnostic variable to address in more general anxiety interventions in ABI.

### Feasibility and Internal Consistency of the IUS-12 in ABI

The low rate of missing items (less than 1%) suggests that the IUS-12 is feasible to use in ABI. A wide range of scores were elicited, indicating no floor or ceiling effects. Furthermore, the IUS-12 demonstrated excellent internal consistency estimates as measured by Cronbach’s alpha, both for the scale total and for the Prospective and Inhibitory subscales. Interestingly, IUS-12 scores were not affected by demographic or ABI characteristics, or whether data were collected before or during COVID-19. This suggests that, as intended, the IUS-12 assesses *responses* to uncertainty following ABI, rather than the *level* of uncertainty.

### Factor Structure of the IUS-12 in ABI

In its initial development, the IUS-12 was thought to comprise two subscales; namely, Prospective Anxiety and Inhibitory Anxiety (Carleton et al., 2007). Broadly consistent with this, in ABI, a confirmatory two-factor model of these subscale scores had a significantly better fit relative to a one-factor model in ABI. However, some fit estimates of this two-factor model (SRMR and RMSEA) were still less than optimal and thus replication of a two-factor model in larger ABI samples may be warranted. It is worth adding that only a subset of possible models was explored here and alternative approaches may be more applicable in ABI (e.g., network factor structures; Bottesi et al., 2020). It may be that in ABI samples, responding tendencies may differ to that of healthy samples (e.g., due to injury-related language impairments affecting interpretation or face validity of the measure; differences in response from acute to chronic stages of ABI; lower divergent validity) and thus result in different factor structures. Factor structure research outside of ABI has noted that a bifactor solution better represents IUS-12 scores (Bottesi et al., 2019; Hale et al., 2016; Lauriola et al., 2016; Saulnier et al., 2019; Shihata et al., 2018; Yao et al., 2021). Notably, not all previous studies have found that Prospective Anxiety items had good loadings in bifactor solutions (Saulnier et al., 2019; Shihata et al., 2018). Finally, critiques of bifactor models postulate that strong theoretical grounds should be established prior to suggesting the use of scale total scores over subscale scores (Decker, 2021).

### Clinical and Theoretical Implications

This study indicates that a well-validated, practical and widely used measure of IU in the general population is feasible and shows strong reliability and validity estimates in ABI. It exhibits the same concurrent and predictive relationships with measures of mood disorder seen in the general population. This opens new methodological seams that could inform improved clinical assessment and better targeted interventions in ABI in which there is a substantially increased risk of mood disorder. In the general population, there have been reported successes in the use of cognitive behavioral therapy in modifying IU (Bomyea et al., 2015; Hebert & Dugas, 2019). There are already indications that in multiple sclerosis, a progressive neurological condition, high IU has been related to maladaptive coping strategies (Alschuler & Beier, 2015) and IU-focused interventions in multiple sclerosis have increased diagnosis acceptance (Molton et al., 2019). While IU may be more obviously relevant to a *condition* that is progressive and therefore uncertain, those with nonprogressive ABIs may still benefit from identifying the “paralysing” effect of uncertainty as a contribution to post-ABI avoidance, generalized anxiety, and social anxiety in intervention settings.

Rather than considering potential modulating effects of progressive versus nonprogressive conditions on IU, “domains” of uncertainty may be worth assessing within interventions, for example, uncertainty such as around one’s identity, perceived functional capacity, ABI recurrence, or changing impairments. Outside of ABI, disorder-congruent situations (e.g., social situations for social anxiety, contamination concerns in obsessive-compulsive disorder) relate more strongly to IU than disorder-incongruent situations (Jensen & Heimberg, 2015). Qualitative themes of uncertainty in ABI (e.g., Salter et al., 2008) may be useful sources for developing IU measures that are sensitive to “uncertainty domains” post-ABI. From a theoretical perspective, the much more challenging question is to what extent IU fits within models of psychological distress post-ABI. It is not yet clear what the distinction is (if any) between increases in the *amount* of uncertainty, the *personal relevance* of sources of uncertainty, and *individual differences* in coping with and processing uncertainty. Distinguishing between these may allow for a better account of how post-ABI distress, specifically anxiety, is generated and maintained.

## Limitations

The study was conducted with participants with chronic ABI; hence, results may differ in acute samples or in progressive neurological conditions. For practical reasons, a portion of study participants self-reported an ABI without requiring evidence of diagnosis, and sampling methods in medical settings may produce different results. The current study was adequately powered to evaluate one- and two-factor analyses, but a larger sample size is required to exhaustively test alternative models of the IUS-12. Parts of the current study took place during the COVID-19 pandemic, and while data presented here suggest that it is a relatively stable measure of trait *responses* to uncertainty, it may be important to take this context into account when the results are compared with those of subsequent studies.

## Conclusion

The IUS-12 is a brief, practical measure of IU that is suitable for use in people with ABI, at least those in chronic stages. The IUS-12 division into Prospective and Inhibitory Anxiety subscales, based on general population studies, formed a better fit to our ABI data than a unitary IU factor, and both subscales had strong reliability estimates over 4 and 20 weeks. There were strong concurrent and predictive validity estimates with respect to mood disorder measures, particularly generalized and social anxiety. Exploratory analyses of other factor solutions for the IUS-12 may be warranted in further ABI studies.

## Supplemental Material

sj-docx-1-asm-10.1177_10731911231182693 – Supplemental material for Measuring Intolerance of Uncertainty After Acquired Brain Injury: Factor Structure, Reliability, and Validity of the Intolerance of Uncertainty Scale–12 Supplemental material, sj-docx-1-asm-10.1177_10731911231182693 for Measuring Intolerance of Uncertainty After Acquired Brain Injury: Factor Structure, Reliability, and Validity of the Intolerance of Uncertainty Scale–12 by Andrea Kusec, Fionnuala C. Murphy, Polly V. Peers and Tom Manly in Assessment

sj-docx-2-asm-10.1177_10731911231182693 – Supplemental material for Measuring Intolerance of Uncertainty After Acquired Brain Injury: Factor Structure, Reliability, and Validity of the Intolerance of Uncertainty Scale–12 Supplemental material, sj-docx-2-asm-10.1177_10731911231182693 for Measuring Intolerance of Uncertainty After Acquired Brain Injury: Factor Structure, Reliability, and Validity of the Intolerance of Uncertainty Scale–12 by Andrea Kusec, Fionnuala C. Murphy, Polly V. Peers and Tom Manly in Assessment

sj-docx-3-asm-10.1177_10731911231182693 – Supplemental material for Measuring Intolerance of Uncertainty After Acquired Brain Injury: Factor Structure, Reliability, and Validity of the Intolerance of Uncertainty Scale–12 Supplemental material, sj-docx-3-asm-10.1177_10731911231182693 for Measuring Intolerance of Uncertainty After Acquired Brain Injury: Factor Structure, Reliability, and Validity of the Intolerance of Uncertainty Scale–12 by Andrea Kusec, Fionnuala C. Murphy, Polly V. Peers and Tom Manly in Assessment

sj-docx-4-asm-10.1177_10731911231182693 – Supplemental material for Measuring Intolerance of Uncertainty After Acquired Brain Injury: Factor Structure, Reliability, and Validity of the Intolerance of Uncertainty Scale–12 Supplemental material, sj-docx-4-asm-10.1177_10731911231182693 for Measuring Intolerance of Uncertainty After Acquired Brain Injury: Factor Structure, Reliability, and Validity of the Intolerance of Uncertainty Scale–12 by Andrea Kusec, Fionnuala C. Murphy, Polly V. Peers and Tom Manly in Assessment

## Notes

1. Based on data from Hale et al. (2016) using a sample of 663 participants, mean communality estimates (correlation of the items to the factor) of the Inhibitory Anxiety subscale and the Prospective Anxiety subscale are 0.69 and 0.56, respectively. Scale total mean communality estimate is 0.61.

2. For the purposes of comparison with predictive validity results, cross-sectional correlations using data from only those who completed all three time points (*N* = 48) were also conducted. The pattern of results remained similar to that presented within the main text in that while both Prospective and Inhibitory Anxiety significantly correlated with all Time 1 study measures, Inhibitory Anxiety had strong correlations overall (as shown in Figure 1).

3. We thank an anonymous reviewer for the inclusion of this point.
