# Supplementary material for: Measuring Intolerance of Uncertainty After Acquired Brain Injury: Factor Structure, Reliability, and Validity of the Intolerance of Uncertainty Scale–12
Source: Assessment. 2023 Jun 26;31(4):794–811. doi: 10.1177/10731911231182693 (PMC11092298; doi:10.1177/10731911231182693)
Supplement: sj-docx-1-asm-10.1177_10731911231182693 – Supplemental material for Measuring Intolerance of Uncertainty After Acquired Brain Injury: Factor Structure, Reliability, and Validity of the Intolerance of Uncertainty Scale–12 [file sj-docx-1-asm-10.1177_10731911231182693.docx]

**Supplementary Materials**

*Distribution of IUS-12 subscales*

A visualisation of the distributions of IUS-12 subscale scores at Time 1 per clinical cut-off on study mood measures is shown in Figure S1. In general, Inhibitory Anxiety seemed to be notably higher in those meeting clinical cut-offs for generalized anxiety, social anxiety, and depression, while Prospective Anxiety levels seemed comparable across probable diagnostic status.

[Figure S1 around here]

*IUS-12 Item Statistics*

IUS-12 scale statistics, including item means and item-subscale correlations, are shown in Table S1. In general, item 2 was rated the highest by ABI participants, while item 6 was rate the lowest. Each subscale item correlated more strongly with the scale total over the opposing subscale.

[Table S1 around here]

*Exploration of Participation Attrition over Time*

Some participant drop-out was expected across time points in the repeated measures design of Study 1. In addition, *apparent* attrition between Time 1 and Time 3 was in fact part of the design. As part of a separate research investigation on IU and COVID-19, *only* those participants who had completed their Time 1 assessment after the onset of the COVID-19 pandemic were invited to take part at Time 3. Hence, the attrition rate across the samples, accounting for this exclusion, was within a range typical of ABI samples.

Potential differences in study outcome measures at Time 1 between those who withdrew versus those retained were explored. There were no significant differences on the IUS-12 subscales, measures of depression, social anxiety, and generalised anxiety, nor were there differences in measures of activity levels, or motivation. On the EBIQ scale, participants who withdrew were more likely to have self-reported higher levels of impulsive behaviour (*t* = -2.36, *p* = 0.02, *d =* -0.46) and greater physical symptoms (*t* = -2.76, *p* < 0.01, *d =* -0.52); otherwise, there were no differences in the remainder of the EBIQ subscales. Given the EBIQ subscales are only used cross-sectionally at Time 1, these were not considered to meaningfully change interpretation of other results concerning IUS-12 reliability, validity and factor structure.

In terms of participant demographics, there was no difference in the proportion of different types of ABIs (e.g., number of participants who had sustained a traumatic brain injury, stroke, tumour, or other ABI) between those who withdrew and those retained (*p* = 0.14). However, those who withdrew had sustained their ABI more recently than those that remained (*t* = 2.43, *p* = 0.02, *d =* 0.52). Whilst this difference is possibly incidental, it is at least plausible that those with more recent injuries had more variable health, had a higher number of competing appointments etc. Participants who withdrew by Time 2 were also more likely to have started the study *before* the onset of COVID-19 (χ^2^ = 37.47, *p* < 0.001). Here, the imposition of lockdowns/social distancing, health concerns, lack of access to the internet or unwillingness to engage in online activity seem likely candidates for some withdrawals. In contrast, those who started *after* the onset of COVID-19, where only online participation was offered, by definition had internet access and were prepared to engage. Otherwise, there were no differences in participant characteristics between those who withdrew and those retained.

Descriptive statistics of self-report measure data between those who dropped out at Time 2 versus those retained are shown in Table S1. In summary, there appears to be some effect of post-ABI symptomology and timeline on attrition though there appears to be no effect on the primary measure of interest – the IUS-12 – suggesting that results are likely robust to differences in characteristics in those retained vs those who withdrew.

[Table S2 around here]
