# Supplementary material for: Measuring Intolerance of Uncertainty After Acquired Brain Injury: Factor Structure, Reliability, and Validity of the Intolerance of Uncertainty Scale–12
Source: Assessment. 2023 Jun 26;31(4):794–811. doi: 10.1177/10731911231182693 (PMC11092298; doi:10.1177/10731911231182693)
Supplement: sj-docx-2-asm-10.1177_10731911231182693 – Supplemental material for Measuring Intolerance of Uncertainty After Acquired Brain Injury: Factor Structure, Reliability, and Validity of the Intolerance of Uncertainty Scale–12 [file sj-docx-2-asm-10.1177_10731911231182693.docx]

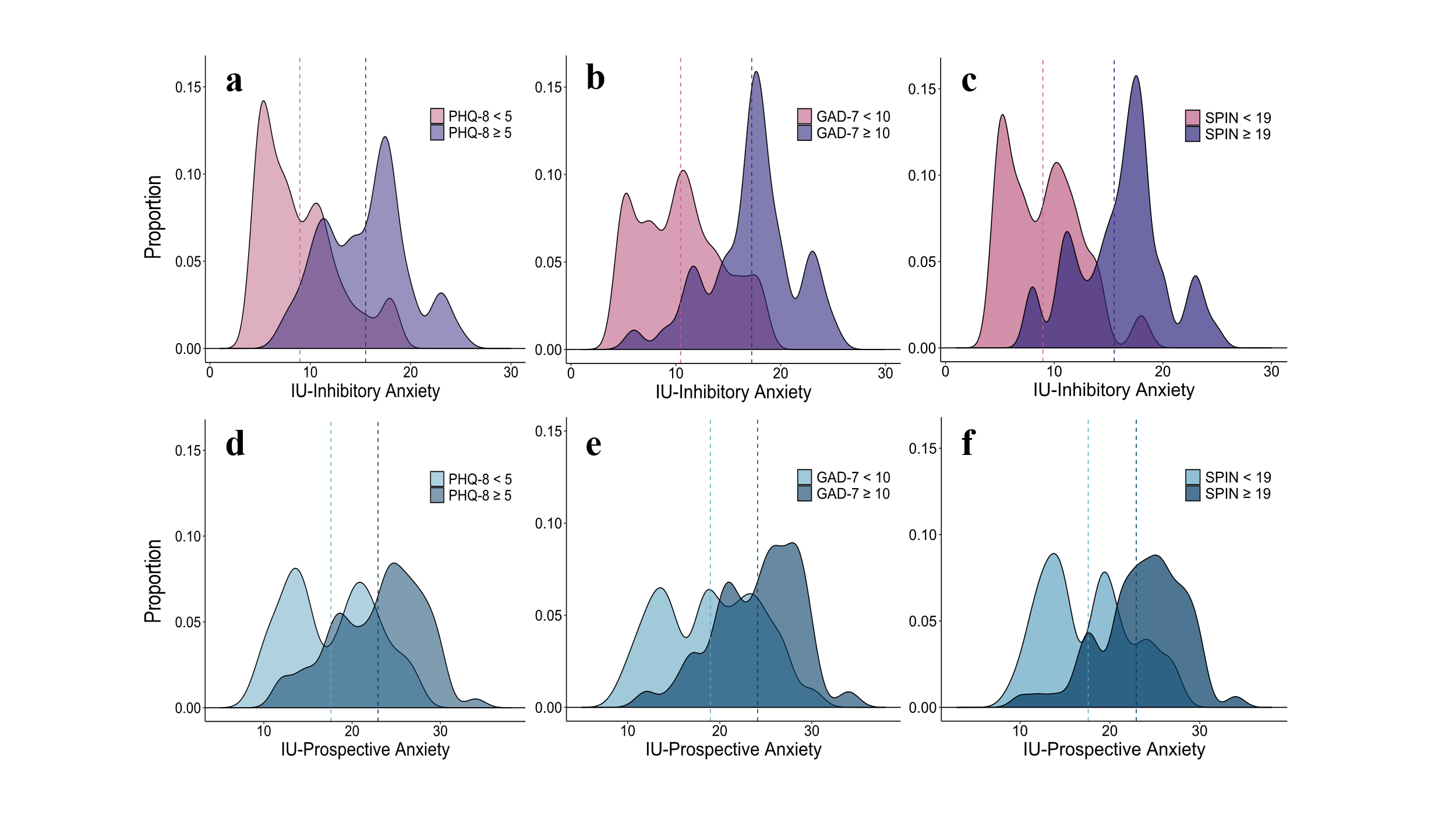


**Figure S1.** Density distributions of IUS-12 Inhibitory Anxiety and Prospective Anxiety scores to depression (PHQ-8, plots a and d), generalised anxiety (GAD-7, plots b and e) and social phobia (SPIN, plots c and f) separated by clinical cut-off scores for each measure (see *Methods* for details on calculations) using Time 1 data (*N* = 118). Group means are visualised along the dotted lines.
