# Supplementary material for: Measuring Intolerance of Uncertainty After Acquired Brain Injury: Factor Structure, Reliability, and Validity of the Intolerance of Uncertainty Scale–12
Source: Assessment. 2023 Jun 26;31(4):794–811. doi: 10.1177/10731911231182693 (PMC11092298; doi:10.1177/10731911231182693)
Supplement: sj-docx-3-asm-10.1177_10731911231182693 – Supplemental material for Measuring Intolerance of Uncertainty After Acquired Brain Injury: Factor Structure, Reliability, and Validity of the Intolerance of Uncertainty Scale–12 [file sj-docx-3-asm-10.1177_10731911231182693.docx]

| **Time 1 Data** | **Retained**  **(*n* = 82)** | **Withdrawn at Time 2**  **(*n* = 36)** | **Cohen’s *d*** |
| --- | --- | --- | --- |
| IUS-12 – *M* (SD) |  |  |  |
| Prospective Anxiety | 20.78 (5.48) | 20.89 (6.17) | -0.02 |
| Inhibitory Anxiety | 12.44 (5.27) | 13.97 (4.87) | -0.30 |
| PHQ-8 – *M* (SD) | 8.78 (6.88) | 9.03 (5.94) | -0.04 |
| GAD-7 – *M* (SD) | 7.86 (6.37) | 7.69 (5.41) | 0.03 |
| SPIN – *M* (SD) | 22.46 (16.30) | 25.76 (19.09) | -0.19 |
| BADS-Activation – *M* (SD) | 19.48 (7.38) | 20.14 (7.08) | -0.09 |
| BADS-Avoidance/Rumination – *M* (SD) | 6.67 (4.90) | 7.64 (4.62) | -0.20 |
| BMQ-Total – *M* (SD) | 79.14 (17.38) | 78.29 (13.01) | 0.06 |
| EBIQ-Somatic – *M* (SD) | 12.39 (3.65) | 12.04 (2.98) | 0.11 |
| EBIQ-Cognitive – *M* (SD) | 19.72 (5.67) | 21.79 (5.66) | -0.37 |
| EBIQ-Impulsivity – *M* (SD) | 15.71 (4.48) | 17.89 (4.87) | -0.46* |
| EBIQ-Communication – *M* (SD) | 6.88 (2.27) | 7.26 (2.13) | -0.17 |
| EBIQ-Fatigue – *M* (SD) | 14.97 (4.42) | 14.44 (3.72) | 0.13 |
| EBIQ-Depression – *M* (SD) | 8.67 (3.29) | 8.56 (2.68) | 0.04 |
| EBIQ-Motivation – *M* (SD) | 8.55 (2.80) | 8.86 (2.93) | -0.11 |
| EBIQ-Isolation – *M* (SD) | 7.17 (2.12) | 6.97 (1.92) | 0.10 |
| EBIQ-Physical – *M* (SD) | 7.11 (2.03) | 8.33 (2.61) | -0.52** |

**Table S2**. Descriptive data of Time 1 self-report measures of those who were retained at Time 2 versus those who withdrew.

IUS = Intolerance of Uncertainty Scale; PHQ-8 = Patient Health Questionnaire; GAD-7 = Generalised Anxiety Disorder-7; SPIN = Social Phobia Inventory; BADS = Behavioural Activation for Depression Scale; BMQ = Brain Injury Rehabilitation Trust Motivation Questionnaire; EBIQ = European Brain Injury Questionnaire.

* *p* < 0.05, ** *p* < 0.01
