# Supplementary material for: Measuring Intolerance of Uncertainty After Acquired Brain Injury: Factor Structure, Reliability, and Validity of the Intolerance of Uncertainty Scale–12
Source: Assessment. 2023 Jun 26;31(4):794–811. doi: 10.1177/10731911231182693 (PMC11092298; doi:10.1177/10731911231182693)
Supplement: sj-docx-4-asm-10.1177_10731911231182693 – Supplemental material for Measuring Intolerance of Uncertainty After Acquired Brain Injury: Factor Structure, Reliability, and Validity of the Intolerance of Uncertainty Scale–12 [file sj-docx-4-asm-10.1177_10731911231182693.docx]

| **IUS-12 item** | **Item**  **Mean (SD)** | **Raw**  **Item-Total Correlation** | **Corrected**  **Item-Total Correlation**^1^ | **Item-Total Prospective Anxiety**  **Correlation** | **Item-Total Inhibitory Anxiety Correlation** |
| --- | --- | --- | --- | --- | --- |
| 1. Unforeseen events upset me greatly. ^a^ | 2.8 (1.2) | 0.75 | 0.73 | **0.73** | 0.65 |
| 2. It frustrates me not having all the information I  need. ^a^ | 3.5 (1.1) | 0.60 | 0.55 | **0.65** | 0.45 |
| 3. Uncertainty keeps me from living a full life. ^b^ | 2.7 (1.3) | 0.75 | 0.73 | 0.60 | **0.81** |
| 4. One should always look ahead so as to avoid surprises. ^a^ | 3.2 (1.2) | 0.61 | 0.57 | **0.71** | 0.42 |
| 5. A small unforeseen event can spoil everything, even with the best of planning. ^a^ | 2.8 (1.3) | 0.72 | 0.68 | **0.69** | 0.63 |
| 6. When it’s time to act, uncertainty paralyses me. ^b^ | 2.4 (1.2) | 0.74 | 0.73 | 0.54 | **0.86** |
| 7. When I am uncertain I can’t function very  well. ^b^ | 2.9 (1.3) | 0.76 | 0.74 | 0.60 | **0.83** |
| 8. I always want to know what the future has in store for me. ^a^ | 3.1 (1.3) | 0.59 | 0.53 | **0.67** | 0.42 |
| 9. I can’t stand being taken by surprise. ^a^ | 2.7 (1.3) | 0.66 | 0.62 | **0.65** | 0.57 |
| 10. The smallest doubt can stop me from acting. ^b^ | 2.7 (1.3) | 0.79 | 0.77 | 0.63 | **0.85** |
| 11. I should be able to organise everything in advance. ^a^ | 3.2 (1.2) | 0.54 | 0.48 | **0.66** | 0.32 |
| 12. I must get away from all uncertain situations. ^b^ | 2.7 (1.3) | 0.76 | 0.73 | 0.64 | **0.78** |

**Table S1**. IUS-12 item statistics (*N* = 176).

^1^Correlation corrected based on item overlap and scale reliability.

^a^Items form part of Prospective Anxiety subscale. ^b^Items form part of Inhibitory Anxiety subscale
